# An overview of graph databases and their applications in the biomedical domain

**DOI:** 10.1093/database/baab026

**Published:** 2021-05-18

**Authors:** Santiago Timón-Reina, Mariano Rincón, Rafael Martínez-Tomás

**Affiliations:** Departamento de Inteligencia Artificial, Universidad Nacional de Educación a Distancia (UNED), C/Juan del Rosal, 16 Ciudad Universitaria, Madrid 28040, Spain; Departamento de Inteligencia Artificial, Universidad Nacional de Educación a Distancia (UNED), C/Juan del Rosal, 16 Ciudad Universitaria, Madrid 28040, Spain; Departamento de Inteligencia Artificial, Universidad Nacional de Educación a Distancia (UNED), C/Juan del Rosal, 16 Ciudad Universitaria, Madrid 28040, Spain

## Abstract

Over the past couple of decades, the explosion of densely interconnected data has stimulated the research, development and adoption of graph database technologies. From early graph models to more recent native graph databases, the landscape of implementations has evolved to cover enterprise-ready requirements. Because of the interconnected nature of its data, the biomedical domain has been one of the early adopters of graph databases, enabling more natural representation models and better data integration workflows, exploration and analysis facilities. In this work, we survey the literature to explore the evolution, performance and how the most recent graph database solutions are applied in the biomedical domain, compiling a great variety of use cases. With this evidence, we conclude that the available graph database management systems are fit to support data-intensive, integrative applications, targeted at both basic research and exploratory tasks closer to the clinic.

## Introduction

Nowadays, the generation, consumption and, more importantly, analysis of highly interconnected data have become ubiquitous. In this situation, where the relationships among data grow both in quantity and in significance, graph models become an appealing solution, as graphs are mathematical entities in which objects are connected. Formally, a graph *G*(*V*, *E*) is composed of an ordered pair of two disjoint sets: vertices *V* (also referred to as nodes) and edges (or links) *E* (1). The graph abstraction directly translates concepts and instances into nodes and their relationships into edges, making it intuitive for data modeling. However, strong graph data is not straightforward in conventional Database Management Systems (DBMSs), and the physical implementation of a given data model and how the relations are treated ultimately depend on the database type.

For example, the basis of Relational Database Management Systems (RDBMSs) are tables (relations) (2–4), where each row represents a single data element of an entity and a single column usually defines a particular data attribute. The standard mechanism to create relationships between entities is by defining unique IDs (primary keys) that can be copied into referencing tables (foreign keys). To exploit these references and include different tables in a database query, the Structured Query Language (SQL) (5) provides the JOIN clause. The relational paradigm is very appropriate for well-defined data structures that are unlikely to change and translate naturally to tables, and the relations among its entities are not numerous and not as relevant as the entities’ attributes. Hence, given its maturity and technological development, RDBMSs are widely used for data storage, with countless examples experienced in everyday life, like user data, inventory tracking, blog posts and many more. However, when most relationships are many-to-many, prevalent in densely connected data, querying the database requires multiple expensive JOIN operations, impacting the performance (6).

Although graphs can be modeled with tables representing vertices and edges, complex queries or graph algorithms (like path traversals) are challenging to optimize without implementing complementary structures, such as adjacency lists (7). These modeling and performance limitations have increased the interest in Graph Database Management Systems (GDBMSs). GDBMSs, in contrast to regular DBMSs, allow working directly with a graph model, avoiding sophisticated engineering to represent relationships efficiently, and provide straightforward ways to store, access and operate graph data, especially for traversing paths and matching subgraphs. Furthermore, the schema-less or schema-optional approach that most GDBMSs follow grants a high degree of flexibility, allowing applications to adapt and evolve quickly and introduce abstraction, specialization of entities and relations among them more easily.

Graph models are present in multiple formal representations and become very powerful when the problem model exhibits varied relations among the entities or concepts. Consequently, the trend in graph databases has permeated into many disparate domains, and we can find applications in Energy Management Systems (EMS) (8), Power Grid Modeling (9) and even less technologically driven fields like Digital Humanities (10). The biomedical domain is a complex area that is inevitably studied in many different sub-domains that are inherently related and connected. For instance, the study of human metabolism requires identifying hundreds of concepts (e.g. metabolites, proteins, complexes and metabolic reaction names) and the relations among them (e.g. consumption, production and catalysis), and graph models provide a valuable framework in this situation. Moreover, the amount of data produced in the ‘omics’ era results in large graphs that become difficult to manage without a database optimized for the task.

**Figure 1.**
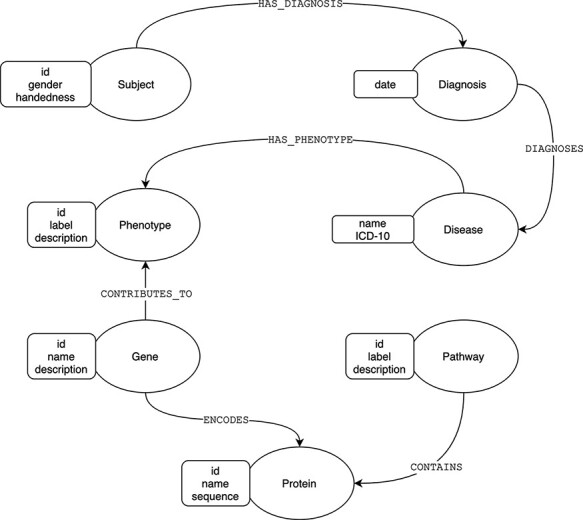
Graph model of diagnoses and its related phenotype–genotype and pathway implications.

**Figure 2.**
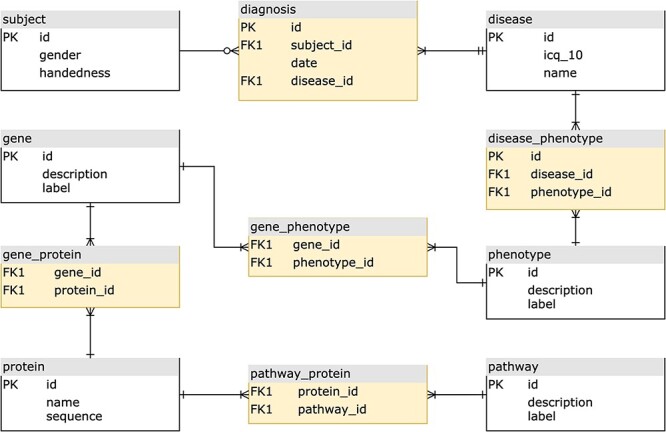
The equivalent normalized relational physical design, with entity tables (white) to store attributes, and join tables (yellow) to implement the relationships.

We can illustrate the differences between the relational and graph-based paradigms depicted in Figures 1 and 2, a stripped-down biological model describing subject diagnoses and their related phenotype–genotype and pathway implications. For most GDBMSs, the physical design resulting from the logical model described in Figure 1 would be almost equivalent. However, in the case of RDBMSs, the implementation from the logical to the final physical design requires dealing with the many-to-many cardinality that most of the model’s relations will have. A typical normalized relational design, at least to the Third Normal Form (3NF) (11), prevents data redundancy by introducing intermediate tables for each relationship between two entities, as shown in Figure 2. For searching heavily connected entities, like genes, this layout would require referencing (joining and sub-querying) several tables multiple times, potentially with various filters, ultimately eroding the query’s performance. Also, complicated queries may end up being rather cumbersome. Thus, designing a relational model for highly interconnected data poses an engineering challenge, especially when the model requires fine-grained semantics, which involves a trade-off between implementing specialized relations (more tables) or limiting the expressiveness at the expense of semantics.

GDBMSs treat relationships as first-class objects, improving the data model’s semantics and easing the adoption of knowledge models and ontologies, which are computer science constructs that provide well-defined vocabularies that allow the precise and machine-readable description of knowledge about a particular domain (12). The biomedical domain has driven and benefited from advances in Knowledge Representation (KR) and storage, being one of the early adopters of ontological research. As a result, there exists a significant number of formal biomedical ontologies (13) that capture and model knowledge from disparate sub-fields, giving rise to initiatives like the Open Biological and Biomedical Ontology (OBO) Foundry (14) and the National Center for Biomedical Ontology (15) to promote harmonization and interoperability. These controlled vocabularies and ontologies support the research in several ways, mainly in data annotation (16–19) and biomedical text mining (20, 21).

In this paper, we survey the adoption of GDBMSs in the biomedical domain to present a summary review from an ‘application perspective’ with categorization and description of biomedical applications employing GDBMSs as storage systems. The applications presented are selected from a broad literature search complying with the following characteristics: (i) are biomedical applications using GDBMSs, (ii) are well documented with papers and websites (iii) have been peer-reviewed. Our coverage of biological graph-powered systems is by no means exhaustive, focusing on recent developments that are high quality, publicly available and expected to be of interest to experts and developers in the community. It is worth noting that, given the overlapping nature of biomedical knowledge, some systems can be classified into more than one category. First, we provide a technological background by exploring the different database models and designs and examining the performance through benchmark studies from the literature. Afterward, we highlight the use of GDBMSs within different applications in a wide variety of biomedical contexts, describing the implications and impact of graph technology in these settings. Finally, we discuss the current state, limitations and possible future lines.

## Background

### Graph database models and design

Graph database models may be defined as those in which the data structures are modeled as a directed, possibly labeled, graph, or its generalizations. The data manipulation is done using graph-oriented operations and type constructors, and appropriate integrity constraints can be defined over the graph structure (22). Over the past decade, graph database implementations have grown from prototypical, application-driven approaches to fully developed products, providing external interfaces, database languages, query optimizers, storage and transaction engines, and management features. This evolution has been actively reviewed (23–28), showing how deficiencies such as the lack of integrity constraints, partition and scalability limitations, or the need for standard graph database languages have been addressed throughout the version history. Besta *et al.* describe the contemporary technological landscape of graph database solutions through a taxonomy of six key design aspects: type of backend technology, data modeling approach, internal data organization, data distribution, query execution and type of transactions (29).

As far as backend technology is concerned, we can see that, at present, most graph database systems are built upon existing storage designs from both relational and NoSQL (30) paradigms, such as key-value, document, wide-column, tuple and object-oriented stores. Key-value stores allocate items as (key, value) pairs, usually in standalone hash tables. Document stores extend key value so that the values are ‘documents’, encoded in standard semi-structured formats such as XML, JSON or BSON (Binary JSON). Wide-column stores represent data through a tabular format of rows with a fixed number of column families (an arbitrary number of columns that are logically related to each other and usually accessed together). Triple stores [also known as Resource Description Framework (RDF) databases] work with the notion of triples (subject–object–predicate), and tuple stores generalize these systems to collect tuples of arbitrary size. Object-oriented stores store data as true objects, identified by object IDs (OIDs) and following a class hierarchy. Using existing engines delivers the advantage of mature and well-tested technology but at the expense of obtaining non-optimized graph data representations and queries. In contrast, native graph databases like TigerGraph (31) and Neo4j are specifically built to maintain and process graphs. Table 1 provides a list of different GDBMSs, which many of the reviewed applications use, with their internal database engines.

**Table 1.** Summary of available implementations by core database engine

Product	Link	Database engine
WhiteDB	http://whitedb.org	Tuple store
GraphDB	https://www.ontotext.com/products/graphdb	Tuple store
OrientDB	https://www.orientdb.org	Document store
ArangoDB	https://www.arangodb.com	Document store
Azure Cosmos DB	https://azure.microsoft.com/es-es/services/cosmos-db	Document store
FaunaDB	https://fauna.com	Document store
RedisGraph	https://oss.redislabs.com/redisgraph	Key-value store
Dgraph	https://dgraph.io	Key-value store
HyperGraphDB	http://www.hypergraphdb.org	Key-value store
MS Graph Engine	https://www.graphengine.io	Key-value store
Titan	https://titan.thinkaurelius.com	Wide-column store
JanusGraph	https://janusgraph.org	Wide-column store
DSE Graph	https://www.datastax.com/products/datastax-graph	Wide-column store
InfiniteGraph	https://www.objectivity.com/products/infinitegraph	Object-oriented store
ThingSpan	https://www.objectivity.com/products/thingspan	Object-oriented store
VelocityDB	https://velocitydb.com	Object-oriented store
Oracle Spatial and Graph	https://www.oracle.com/technetwork/database-options/spatialandgraph/overview/spatialandgraph-1707409.html	RDBMS
Sparksee/DEX	http://www.sparsity-technologies.com	Native graph database
TigerGraph	https://www.tigergraph	Native graph database
GraphBase	https://graphbase	Native graph database
Memgraph	https://memgraph.co	Native graph database
Neo4j	https://neo4j.com	Native graph database

Regarding data modeling, Labeled Property Graphs (LPG) and RDF are the most common graph models found in graph database systems (32–34). LPG augments the simple graph model to allow defining labels for nodes and edges, as well as an arbitrary number of properties (also called attributes) for both. RDF, a World Wide Web Consortium (W3C) standard, was conceived as a collection of specifications for representing information to allow easy data exchange between different data formats, and graphs arise from the collection of triples in the form of subject, predicate and object (s, p, o). The RDF format is widely used in biomedical setups, due mainly to the fact that RDF is a serialization and data instantiation format for OWL-based bio-ontologies, and new systems using native graph databases rely on transformations between models to fully exploit their features.

**Figure 3.**
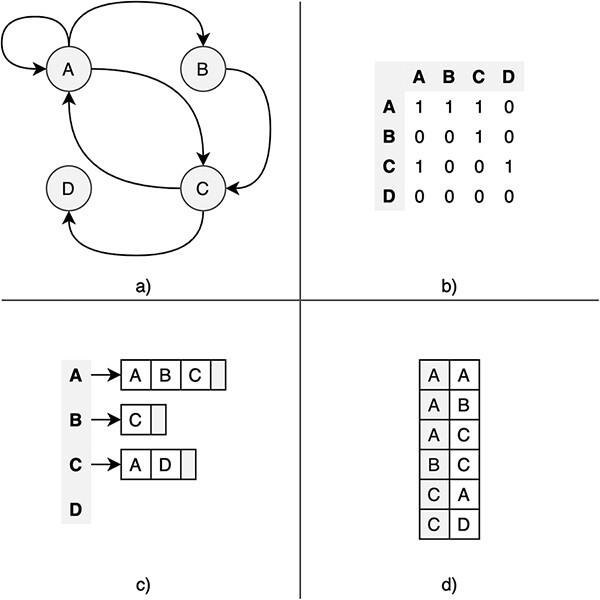
Graphic description of the most common graph representation formats. (a) Original directed graph; (b) adjacency matrix; (c) adjacency list; and (d) edge list.

Likewise, systems need to define data structures to represent graphs in the storage layer. The most common representation formats are the adjacency matrix (AM), the adjacency list (AL) and the edge list (EL). Figure 3 shows a graphical representation of these formats. The AM is a square matrix where its cells indicate whether vertex pairs are adjacent (connected) or not. In the AL format, each vertex has an associated adjacency list containing the IDs of all adjacent vertices. The difference with EL is that AL explicitly stores edges with its source and destination vertex. The AL format is efficient on traversal operations, and many graph databases use it. Other features, such as index support, are also relevant for the overall performance.

Data distribution may be achieved through data replication or sharding. With replication, each instance maintains a copy of the dataset, while sharding fragments the data across instances. Distribution becomes essential when dealing with large amounts of data, and query execution is directly linked to it. Multi-server query execution can be enabled in several ways. The concurrent execution allows the execution of different queries at the same time, providing higher throughput. With parallelization, a single query can be executed across servers to obtain lower latencies. Because managing large amounts of data can compromise the system’s performance or availability, these features can become essential for projects in this situation.

Finally, GDBMSs can be evaluated by the support of transactions. Specifically, Atomicity, Consistency, Isolation, Durability (ACID); Online Transaction Processing (OLTP); and Online Analytics Processing (OLAP) support. OLTP systems focus on smaller transactional queries, while OLAP systems execute more expensive analytic queries that span whole graphs.

The literature reveals that the field is evolving rapidly and many referenced databases have either already been discontinued or greatly improved at the time of writing.

### Performance and benchmarking

Because of their innate capabilities in dealing with highly interconnected data, graph databases have been attracting attention in the past years. As different technological implementations of graph database engine have emerged, so has the need for accurate, quantitative performance comparisons between them by using standardized queries and workloads. Furthermore, the differences in relational and graph-based paradigms also raised questions about how they would behave in different contexts. Table 2 summarizes the surveyed benchmark studies.

**Table 2.** Relevant benchmarking studies

Reference	Benchmark/methodology	GDBMS	RDBMS/NoSQL	Description
(42)	Own implementation	Neo4j	MySQL	Technology comparison about recording and querying data provenance information. Executes objective benchmarks to measure query response time and disk space usage. Also, it provides subjective comparisons based on system documentation and usage experience. Concludes that Neo4j outperforms on structural queries, but it is premature to use a graph database in production environments for data provenance
(57)	HPC Scalable Graph Analysis Benchmark	Neo4j Jena HypergraphDBDEX		Evaluates the performance of selected systems with the HPC benchmark. This benchmark employs R-MAT (58) for graph generation and measures the execution time over different kernels: data loading, scan edges, 2-hops subgraph building and Traversed Edges Per Second. All four platforms perform well on small graphs. Only DEX and Neot4j were able to load the largest graphs. DEX showed the best performance
(47)	Own implementation of a small social network–like problem	Neo4j	MySQL	Small comparative analysis with social network queries. In this study, Neo4j outperforms MySQL in all queries
(59)	GDB, an extensible tool to compare Blueprints-compliant graph databases	Neo4j DEX Titan OrientDB		A Tinkerpop-based distributed benchmarking framework to compare Blueprints-compliant graph databases. The benchmark measures traversal, load and intensive workloads. The results show that all databases perform similarly on read-only operations, while Titan and DEX stood out on read–write workloads, and Neo4j did on traversal workloads. Code available at https://github.com/Jsalim/GraphDB-Benchmark
(43)	Bioinformatics graph processing problems	Neo4j	PostgreSQL	A query benchmark that evaluates Neo4j against PostgreSQL in typical bioinformatics graph processing problems. The study employed the human interaction network from STRING v9.05 (60) and measured the response time for finding immediate neighbors and their interactions, finding the best scoring path between two proteins and finding the shortest path between them. Neo4j outperformed PostgreSQL, showing speedups of 36× (immediate neighbors), 981× (best scoring path) and 2441× (shortest path)
(44)	Graph-based extension to Conditional random field Protein–Protein Interface identification	Neo4j	Microsoft SQL Server	A case study on how Neo4j can be applied to the bioinformatics problem of protein–protein interface identification
(45)	Biomedical graph traversal operations	Neo4j	MySQL	Compares the performance by employing biological network information from 21 different datasets and ontology resources. The benchmark measured the query response time of retrieving all data that traverse the relationships among genes, drugs and diseases that increased the expression of the BRCA1 gene. The results report that Neo4j outperformed MySQL in all cases and highlights the importance of system tuning to obtain better performance
(49)	LDBC SNB	Neo4j		Analyzed the fundamental points of graph databases and employed the LDBC-SNB to evaluate the performance of Neo4j. Without much detail, the work concludes that Neo4j shows acceptable behavior when dealing with different sizes of graph databases.
(55)	Own implementation of comparative measures over a medical care setup	Neo4j	Oracle	Compares the performance of Oracle 11g and Neo4j over a hospital health-care system use case with a set of predefined queries with different join/subquery requirements. Neo4j outperformed Oracle in 4/5 of the queries.
(50)	Extension of the LDBC SNB IW to simulate real-time transactional workloads	TitanDB Neo4j Virtuoso	PostgreSQL	An improved graph database benchmarking architecture for real-time transaction processing built upon LDBC-SNB and Apache Kafka (https://kafka.apache.org/). Provides LDBC-SNB reference implementations for Gremlin (61), SQL and Cypher. The experiment employed two synthetic datasets with scale factors of 3 and 10, to execute read-only graph queries (point lookups, one-hop traversals, two-hop traversals and single-pair shortest path) and simulate a real-time Interactive Workload. Their results showed that Neo4j achieved higher throughput than TitanDB and that PostgreSQL provided the best overall performance followed by Virtuoso (https://virtuoso.openlinksw.com/) (SQL mode). Concludes that RDBMSs with a native SQL interface provides the best performance under real-time streaming scenarios. Gremlin Server incurs significant overhead
(56)	Follow-up of Khan 2017 with database tuning	Neo4j	Oracle	Follow-up work of Khan 2017 where they improve the performance of Oracle 11g database about 35% by creating separate tablespaces for each schema and table, and five more query workloads. Still, despite the physical tablespace tuning technique of Oracle 11g, Neo4j outperforms it in all proposed scenarios
(52)	Domain-agnostic workloads. Two-way adaptations to compare graph databases with other implementations. TPC-H and LDBC	Neo4j ArangoDB	MySQL Microsoft SQL Server Oracle PosgreSQL RocksDB HBase Cassandra	Comparative evaluation between RDBMSs and GDBMSs under a unified benchmark that extends the TPC-H standard RDBMS benchmark and LDBC. The query workload consists of three main categories: atomic relational queries (projection, aggregation, join and order by), TPC-H query workloads, and five graph algorithms from LDBC. The metrics measured the average query processing time, memory usage (peak) ratio and CPU usage (peak) ratio of five query runs. This benchmark concluded that RDBMSs outperform GDMBSs by a substantial margin under the workloads that mainly consist of group-by, sort and aggregation operations. On the other hand, GDMBSs are superior in the execution of those workloads that mainly consist of multi-table join, pattern matching and path identification
(51)	Complete LDBC-SNB implementation for Neo4j and TigerGraph	Neo4j TigerGraph		A complete implementation of the LDBC-SNB benchmark in Neo4j and TigerGraph native GDBMSs. The experimental setup consisted of four scale factors that ranged from 1 GB to 1 TB deployed on three computing architectures. The results can be fairly summarized in three key points: TigerGraph stores graph data considerably more compactly than Neo4j, Neo4j is faster at ingesting raw data than TigerGraph, and lastly, Neo4j is faster than TigerGraph only in 13 of the 368 configurations. Concludes that TigerGraph is superior to Neo4j on the LDBC-SNB benchmark
(46)	Own benchmark implementation with biomedical data. Data loading, path traversal and aggregation tests	Neo4j		Evaluates the aptness of the database system in terms of analysis and visualization of a GRN by measuring three test cases (bulk data insertion, path queries and aggregation queries) with a small and large dataset. The results showed that Neo4j performed well in most of the tests; after warming up the cache, the performance improved drastically, reducing query time by about 64% for both dataset sizes. In the same vein (44), the queries that involved more edge operators performed worst
(62)	TigerGraph’s benchmark	RedisGraph TigerGraph Neo4j Neptune JanusGraph ArangoDB		The study executes TigerGraph’s benchmark to evaluate RedisGraph against leading graph databases. Using graph data from Twitter and Graph500 generator, the benchmark measures the query response time for *k*-hop neighborhood count, *k* = 1,2,3 and 6. RedisGraph outperforms all competitors, and the study highlights additional opportunities for enhancement: aggregations, enhanced GraphBLAS (http://graphblas.org), Cypher clauses/functionality to support more diverse queries

Within standard benchmarks, the Linked Data Benchmark Council (LDBC) (35) is one of the most consistent works in this topic, and its workloads have been employed and adapted in many benchmarking studies. The library currently includes three kinds of workloads: interactive, business intelligence and graph analytics. Interactive workloads focus on general graph database operations, executing read-only (short and complex) and transactional update queries. Business Intelligence workloads are designed to stress different performance aspects, employing read-only aggregation operations over significant volumes of data that span large parts of the graph. The last workload, ‘graphalytics’ (36), proposes six graph algorithms to enable the objective comparison of graph analysis platforms: Breadth-First Search (37), PageRank (38), weakly connected components (39), community detection using label propagation (40), deriving the local clustering coefficient (41), and computing single-source shortest paths.

GDBMSs have been assessed in studies from different contexts, like data provenance (42), biomedical settings (43–46) and social networks (47–52). Most of the social network benchmarks use or adapt the LDBC’s Social Network Benchmark (SNB) (53). In parallel with technological surveys, these studies show how GDBMS technology has matured and grown into a competitive and heterogeneous environment, with its weaknesses and strengths.

The number of edges involved in a query has a big impact on performance (44, 46). Likewise, subgraph-matching queries are more challenging to handle in large datasets, in contrast to traversal queries employed in some of the works. Lastly, GDBMSs are, in general, less optimized for aggregate operations (25, 51, 52, 54). In contrast, all the studies acknowledge that schema-less provides a high degree of flexibility to accommodate new nodes or relations, avoiding the need to restructure the schema. GDBMSs are more efficient traversing large graph instances, with lower computational cost than RDBMSs (42, 43, 45, 47, 52, 55, 56), because the search space is reduced to directly connected nodes, avoiding scanning the entire graph to find the nodes that meet the search criteria. Furthermore, graph algorithms (e.g. pathfinding, community detection, centrality or similarity) are more natural to implement and even available out of the box, like the case of Neo4j’s Graph Data Science Library (https://neo4j.com/graph-data-science-library/) or TigerGraph’s (31) GSQL Graph Algorithm Library (https://docs-beta.tigergraph.com/tigergraph-platform-overview/graph-algorithm-library).

To compare different paradigms, benchmarking implementations require an extra effort to address peculiarities. In the case of RDBMSs vs. GDBMSs (52), Cheng *et al*. propose a unified benchmark that extends the TPC-H (http://www.tpc.org/tpc_documents_current_versions/pdf/tpc-h_v2.18.0.pdf) standard RDBMS benchmark and LDBC using transformation mechanisms between relational and graph data, making it possible to evaluate different systems on the same datasets, query workloads and metrics. The query workloads consist of three main categories. Firstly, atomic relational queries (Projection, Aggregation, Join and Order by) aim to evaluate the performance of primitive relational operations implemented in GDBMSs. Secondly, TPC-H query workloads evaluate the performance of GDMBSs on operations that legacy RDBMSs perform well. And lastly, graph query workloads composed of five graph algorithms in the LDBC Benchmark aimed to evaluate the performance of RDBMSs under the situations GDBMSs are supposed to be efficient.

Nevertheless, on the dichotomy between RDBMSs and GDBMSs, we find how late benchmarks show equivalent or even better performance of the former in different settings, questioning whether it is appropriate to favor GDBMSs over RDBMSs without a proper evaluation of the context. We can find one example in real-life high-throughput scenarios, like those with critical concurrent access (59) or streaming transactional workloads (50), where GDBMSs are less prevalent. In these settings, RDBMSs can deliver competitive performance for OLTP-like online social networking applications, especially in single-node setups. Moreover, the implementation and optimization of graph analytics in RDBMSs are growing areas of research (63–66).

The physical data persistence strategy impacts the overall performance in both paradigms. For example (50), Pacaci *et al*. show how similar SQL queries over the same database schema drive different performance in PostgreSQL and Virtuoso (SQL). The difference is attributable to the fact that Virtuoso employs columnar storage, which is known to suffer under transactional workloads with frequent updates, while PostgreSQL implements row-oriented storage. In the case of GDBMSs, adjacency lists are common in native graph storage, as they enable index-free adjacency access and provide apparent advantages for read operations. However, other storage approaches offer better performance regarding write operations, as is the case of key-value storage engines implementing the LSM-tree (67) index. Moreover, tuning procedures are of utter importance to achieve the best possible performance regardless of the system, like optimizing indexing or tablespaces, as some studies report.

## Graph database applications in the biomedical domain

Biomedical research produces large amounts of densely interconnected data belonging to many different domains, and storing such data has always presented a technological challenge. Storing graphs using traditional relational databases presents several drawbacks. Relational databases rely on fixed schemas and usually require redesigns when introducing new data structures, affecting flexibility, efficiency and scalability. More generic data models would require many intermediate tables to represent many-to-many relationships, degrading the overall performance because of the need for multiple join operations to traverse interconnected networks. As graph databases matured, they started to gain more attention in the bioinformatics community, given the ubiquity of graphs in this domain. Consequently, many tools emerged to interoperate between formats and paradigms. Table 3 brings together some of the most relevant ones.

**Table 3.** A short list of useful graph-oriented open-source tools and utilities

Tool	Reference	Code	Description
STON	(74)	https://sourceforge.net/projects/ston	Java-based framework for transforming SBGM models to graphs
Pheno4J	(75)	https://github.com/phenopolis/pheno4j	Java library to load patient genetic variants and phenotype data into Neo4j
Recon2Neo4j	(76)	https://github.com/ibalaur/Recon2Neo4j	Java library that allows loading SBGM models into Neo4j and parsing for translating the Neo4j JSON networks into SBML and SIF formats
ANIMA	(77)	https://github.com/adeffur/ANIMA	R framework for producing multiscale association networks, loaded in Neo4j
SciGraph		https://github.com/SciGraph/SciGraph	Neo4j backed ontology store
Dipper		https://github.com/monarch-initiative/dipper	Python package to generate RDF triples from common scientific resources. Includes mappings and parsers for many sources from different domains
NSMNTX		https://github.com/neo4j-labs/neosemantics	Neo4j plugin that enables the use of RDF in Neo4j
Tarql		https://github.com/tarql/tarql	Java and Apache ARQ based command-line tool for converting CSV files to RDF using SPARQL 1.1 syntax
RDF2Neo	(78)	https://github.com/Rothamsted/rdf2neo	Java-based project providing configurable components to convert RDF data into Cypher commands that can populate a Neo4j graph database

The evolution of Knowledge Representation technologies and, more specifically, ontology languages like OWL, enables more complex and interconnected models. Although many of these tools do not necessarily use an explicit graph model, it is commonly implicit in the semantics, opening the door to exploit graph features. One remarkable example of this approach is the Open Biomedical Ontologies (14), which many of the works we are about to describe employ as foundational models. Table 4 summarizes publicly available graph-powered systems.

**Table 4.** Publicly available graph-powered Biomedical data systems

Platform	Reference	Domain/scope	Implementation	Interfaces	Database
Arena-Idb	(79)	Genetics			Hybrid MySQL-Neo4j
cyNeo4j	(68)		Cytoscape App	Cytoscape GUI	
HRGRN	(80)	Genetics Metabolomics	Web platform	Web	
miTALOS	(81)	Pathway analysis	Web platform	Web	
Biochem4j	(82)	Biochemistry	Exposed database	Neo4j browser REST	Neo4j
Recon2Neo4j	(83)	Metabolomics Proteomics	Exposed database	Neo4j browser	
GeNNet	(84)	Transcriptome analysis	Local web platform	Web Database interface	
Monarch Initiative	(85)	Phenotype–Genotype analysis		Web Data endpoint Ontology endpoint	
Reactome	(86, 87)	Molecular biology Pathway analysis	Web platform	Web Cypher interface REST	
Spfy	(88)	Bacterial WGS	Web platform	Web	Hybrid Blazegraph-MongoDB
GREG	(89)	Genetics	Web platform	Web Cypher interface	Neo4j

### Applications in systems biology

Intrinsically, systems biology models encode networks of entities and biological processes, such as reactions. As advances in molecular biology produce more extensive and complex networks, the computational demand for analyzing those increases drastically. Consequently, the use of in-house software and desktop solutions started to become a bottleneck. GDBMSs allow decoupling a significant part of the computational needs to dedicated server machines, providing improved tuning of resources for optimal query and algorithm execution performance. One good example is cyNeo4j (68), a Cytoscape (69, 70) app to link this popular network analysis desktop program to a server environment using Neo4j. It enables the user to upload network data and run algorithms both locally and on the Neo4j server, creating an interactive workflow that uses the computational strength of the Neo4j server without interrupting the typical workflow in Cytoscape.

Standard formats of the domain, like Systems Biology Markup Language (SBML) (71) or CellML (72), enable modeling biological systems in terms of functional, behavioral or structural aspects, including meta-data and semantic annotations to relate model entities to external resources describing the underlying biology. These meta-data are of great importance to facilitate model reuse and reproducibility, but this introduces heterogeneity, which complicates the design in fixed-schema database systems (73). Henkel, Wolkenhauer and Waltemath employed Neo4j to store SBML and CellML models, including ontology terms and relations from the semantic annotations that these formats support, effectively combining computational models, semantic annotations and simulation experiments. The approach integrated widely adopted bio-ontologies, adding all classes and relations as nodes and edges but leaving out cross-references between concepts of different ontologies. This integration allows querying the information hidden in the semantic annotations of in-model representations and simulation descriptions. Furthermore, it allows defining flexible connections between the data domains, incorporating links between annotations, whole models and model entities.

The Systems Biology Graphical Notation (SBGN) (90) is another standard for visual representation of biological networks. It is composed of three orthogonal languages for representing different views of biological systems: Process Descriptions (PDs), Entity Relationships (ERs) and Activity Flows (AFs). SBGN-to-Neo4j (STON) (74) is a Java framework to transform SBGN markup language files into a Neo4j graph representation, focused only on the PD and AF sub-languages. The authors report that the persistent graph representation yields several benefits, e.g. efficient management and querying of networks, identification of subgraphs in networks, merging of SBGN diagrams/existing pathways into more extensive systems, or the comparison of different layers of granularity in SBGN languages.

### Applications in biological and medicinal chemistry

The fields of Biology and Biochemistry have been a pioneer in the development of new data standards and knowledge representation paradigms, such as ontologies, to foster reuse, integration and translation of research data. These standards enable publicly available data resources such as UniProt (91), KEGG (92) and NCBI Taxonomy (93) to soft-link entities between each other, allowing the user to follow such links by manual browsing or through specialized workflows. The introduction of graph databases made it easier to integrate these resources explicitly. Built on Neo4j, Biochem4j (82) provides an integrated, queryable database that warehouses chemical, reaction, enzyme and taxonomic data from ChEBI (94), MNXref (95), Rhea (96), KEGG, UniProt and the NCBI Taxonomy resources. Biochem4j translates ontology entities and raw biological data into an integrated graph representation, which, leveraged through Cypher query language, allows performing queries and detecting patterns across the whole range of available information.

Logically, graph representations apply to lower-level chemistry and related fields, like drug discovery research. One example is the fragment-based drug discovery (FBDD) (97), in which the validation stage of a project involves testing sensible close analogs of a fragment hit. This process needs adequate search tools to mine the many millions of similar compounds that are currently available in the fragment space from corporate collections or commercial suppliers. The Fragment Network (98) employs Neo4j to allow the user to search the chemical space around a compound of interest. The graph model treats each compound as a set of rings, linkers and substituents, with a resulting network containing a total of 23 million nodes and 107 million edges.

### Applications in the omics domain

In the last five years, the usage of graph databases to support the integration of genomic, proteomic, metabolomic and phenotypic data has substantially increased. Most of the authors conclude that GDBMSs are valuable tools to deal with heterogeneity and lax structured data models because these provide a high degree of flexibility and lay the foundations for building integrated solutions.

#### Biological pathways

Repositories of metabolic maps, reconstructions, pathways and interactions provide fundamental tools for the biomedical investigation. Examples of these repositories are the Reactome Knowledgebase (99), Recon2 (100) and the latest development, Recon3D (101).

Reactome is a comprehensive repository of molecular reactions that include signal transduction, transport, DNA replication, protein synthesis and intermediary metabolism. Reactome contains a detailed representation of cellular processes, as an ordered network of molecular reactions, interconnecting terms to form a graph of biological knowledge. This structure serves both as an archive of biological processes and as a tool for discovering unexpected functional relationships in data. Reactome’s data model initially follows a frame-based design stored in a relational MySQL database. Overcoming the relational model’s intrinsic limitations requires an increased level of abstraction in its physical design to accommodate new concepts, ultimately affecting query complexity and execution time. As graph database systems have matured, the limitations of storing pathway data in relational databases have become more evident, motivating the project to develop tools to migrate the content into a Neo4j database (86, 87). The Reactome case is especially relevant because it exhibits a detailed description of the process to adopt a native graph database and how it improved the performance and capabilities of the whole system. On the one hand, the average query time dropped from 173.11 ms to 12.56 ms, a 93% reduction. On the other hand, the new graph model provides more straightforward ways to perform complex queries over metabolic pathways.

Recon2 is another large community-driven reconstruction of the human metabolic network, with thousands of reactions, unique metabolites and proteins, included in an SBML model. A model of this size and complexity comprises a challenge for advanced exploration involving associations between multiple concepts (e.g. network neighborhood of metabolites, shortest pathways between metabolites, proteins and complexes). Recon2Neo4j (76) is a Neo4j-based metabolic framework that models relevant concepts involved in the metabolic reactions as nodes in the graph database and the relationships among them as connecting edges, facilitating the exploration of comprehensive and highly connected human metabolic data and identification of metabolic subnetworks of interest.

HRGRN (80) is an integrative database for plant signal transduction, metabolism and gene regulation networks that is also backed by Neo4j. The solution, implemented as a web platform, provides the user with a graph-centered search interface to explore these biological systems, allowing to find potential paths or build either node-centralized or nodes-of-interest subnetworks. Regarding the data model, it followed an *adhoc* approach, where biological entities (such as genes, proteins, small compounds and RNAs) are represented as nodes. For the relations between these entities, they defined eight types of edges that link the above nodes based on their biological functions. The Property Graph model is employed to attach a property indicating whether the relationship was validated or predicted.

BioGraphDB (102) is a bioinformatics database to combine different types of data from ten online public resources related to genes, microRNAs (miRNAs), proteins, pathways and diseases. To integrate these disparate resources, it builds on an Extract-Transform-Load (ETL) ecosystem capable of dealing with several formats (Tab delimited, XML, EBML and SQL) with a precise execution order to satisfy dependencies between the integrated resources. This process maps each biological entity and its properties into a vertex and its attributes, and relationships between two biological entities into edges. In this case, the GDBMS of choice was OrientDB. When operating in graph mode, referenced relationships are like edges, accessible as first-class objects having start and end vertices and properties. This feature allows representing a relational model as a document-graph model, maintaining the relationships. With the end-user in mind, the Biograph web application (103) allows users to query, visualize and analyze biological data belonging to the sources available on BiographDB. However, the system is leaned toward a technical, graph knowledgeable audience, with explicit Gremlin query interfaces.

Similarly (104), Lysenko *et al*. illustrate how to build a graph structure to relate biomedical information at different levels and provide biological context to disease-related genes and proteins. It integrated genomic and proteomic data along with disease concepts to investigate possible relations between specific protein interactions, pathways, and typical phenotypes associated with asthma disease. In this case, the modeling strategy follows a protein-centric approach without a rigid schema or upper model (such as an ontology). This approach provides a higher degree of flexibility to integrate many semi-structured data sources and eases the development of *adhoc* solutions, but at the expense of data standardization. The study provides a good insight into how graph databases can facilitate hypothesis generation. Another relevant contribution is to show how targeted Cypher queries exploit known structures, as well as graph algorithms like network neighborhood analysis, to provide biological context. An example of structural queries is obtaining proteins common to asthma and other related respiratory diseases, where protein nodes are connected to health conditions with a concrete ‘associated’ relation. They also demonstrate how simple graph traversal queries have the potential to assist in hypothesis generation by exploring relationships between concepts. For instance, to explore the relationship between asthma and alterations in circadian rhythm, they identify all shortest paths in the graph between asthma disease and a subset of protein-coding genes that generate and regulate circadian rhythms.

#### Epigenetics

Epigenetics is a growing area of research within the biomedical domain, and it is being used in many different contexts, such as the study of cancer. Existing relational databases that focus on various features of cancer pathways are restricted because the integration of multiple data types in relational databases is nontrivial, and the concept linking needed in the exploration of cancer-related hypotheses is limited. EpiGeNet (83) is a graph database that stores conditional relationships between molecular (genetic and epigenetic) events observed at different stages of colorectal oncogenesis. It integrates statistical data on molecular interdependencies recognized in colorectal cancer development, mined from StatEpigen (105) (a manually curated and annotated database) into a Neo4j instance. For the data model, ‘MolecularEvent’ nodes represent molecular events of conditional relationships, modeled as edges in the graph. The edge type is determined by phenotype information and the direction by the conditionality of the relationship. Attributes of ‘MolecularEvent’ are used to store event type and gene information, and the probability value is stored as a property of the edge. The resulting graph makes it possible to explore path connections associated with the highest ‘incidence score’ and employ Cypher queries in tasks like identifying genetic–epigenetic modifications, or molecular phenomena observed and reported in the specialized literature.

#### Transcriptomics

The transcriptome is the complete set of all RNA molecules in a cell, a population of cells, or in an organism (106). Transcriptomics studies generate large amounts of data, raw or processed, that may be deposited in public databases to make them available for a broader scientific community (107). These data can be expressed as gene expression and interaction networks, which may additionally be integrated with other biological datasets, such as protein–protein interactions (PPIs), transcription factors (TFs) and gene annotations. In this context and to evaluate the performance of Neo4j (46), Wiese *et al*. constructed Genome Regulatory Networks (GRNs) based on known enhancer–promoter interactions (EPIs) and their shared regulatory processes by focusing on cooperative TFs. Exploiting these data, we can find platforms like the non-coding RNA Human Interaction Database (ncRNA-DB), later evolved into Arena-Idb (79), miTALOS v2 (81), GeNNet (84), the Association Network Integration for Multiscale Analysis (ANIMA) (77) and the Gene Regulation Graph Database (GREG) (89). Except ncRNA-DB, all these platforms employ Neo4j as the GDBMS.

The ncRNA-DB is built on top of OrientDB, which translates class instances into nodes, permitting to follow an object-oriented design consisting of four main classes and its specializations: BioEntity, Alias, DataSource and Relation. The database imported and integrated associations among non-coding RNAs (miRNAs, circulating miRNAs, Long non-coding RNAs (lncRNAs) and other non-coding RNAs), genes, RNAs and associated diseases from 10 online databases. ncRNA-DB provides three alternative interfaces: a Cytoscape app named ncINetView, a web interface, and a command-line interface for raw resource queries. Later, ncRNA-DB evolved into Arena-Idb, introducing several improvements like a mapping procedure for managing entities, an accurate integration process or reconstructed data storage. The updated dataset included seven new sources [such as Disease Ontology (108), lnc2cancer (109), lncACTdb (110), PSMIR (111), StarBase (112) or TarBase (113)]. Arena-Idb follows a hybrid RDBMS and GDBMS implementation by using MySQL to store names, annotations and sequences and Neo4j to handle the construction and visualization of the networks of thousands of biological entities.

To provide a tool to identify pathways regulated by miRNAs in a tissue-specific manner, miTALOS v2 employs Neo4j to integrate several heterogeneous data sources and directly model molecular entities and their interaction networks. This graph model represents miRNAs, genes, pathways and tissues as nodes. miRNAs are connected to genes with ‘REGULATES’ relationships, genes to tissues with ‘EXPRESSED’ and genes to pathways with ‘MEMBER’ relationships. The graph structure allows to, for instance, query the target genes of a miRNA expressed in a tissue or the pathways in which the target genes are involved. Furthermore, the schema-less approach enables the platform to keep updated and integrate new aspects like lncRNAs as regulators of gene expression or disease-specific expression profiles to extend tissue-specific gene expression.

GeNNet is an integrated transcriptome analysis platform that unifies scientific workflows with graph databases for selecting relevant genes according to the evaluated biological systems. The framework consists of three main components: the Scientific Workflow (GeNNet-Wf), the Graph database (GeNNet-DB) and the web interface (GeNNet-Web). GeNNet-DB uses an in-house data model to group nodes and edges into classes, according to the nature of the objects [e.g. GENE, BP (Biological Process), CLUSTER, EXPERIMENT and ORGANISM], and preloads a set of specified organisms to serve as the initial layout. Along with other associated elements, it includes genes annotated/described from ENTREZ (114) and their relationships integrated from STRING-DB (60), which contribute to posterior transcriptome analysis. The study provides analyses from the hepatocellular carcinoma (HCC) use case, demonstrating how concise graph operations through Cypher queries are capable of solving relatively complex topological questions, like finding the most connected genes that establish known connections to the PPI network. These genes act as hubs and may be associated with relevant pathways in the experimental context.

ANIMA allows the summarization and visualization of different views of the state of the immune system under different conditions and at multiple scales. The framework generates a multiscale association network from multiple data types by executing a comprehensive analytic workflow, enumerating bipartite graphs from the results and merging all graphs into a single network in Neo4j. ANIMA is architectural and conceptually similar to GeNNet, differing mainly in the detail of the implementation, the containerization approach, and the complexity of the model.

GREG is an integrative database that merges numerous source databases providing different scopes (e.g. DNA–DNA interaction, PPIs, bindings, DNA annotations or human cell data). It follows an in-house data model and takes advantage of the graph model to tackle challenges like integrating EPIs (with DNA binning strategy) or harmonizing data from chromatin interaction technologies with very different resolutions. When using small bins, its graph comprises more than 2 M nodes and more than 19 M edges, and the main limitation is that, due to include all non-coding regions, search time grows with the size of the genomic range. GREG provides both direct access to the Neo4j (via Cypher) and a friendly web platform. Through the web interface, the user can specify search parameters and access typical network analysis algorithms.

### Biological knowledge graphs

While there exist multiple definitions of Knowledge Graphs (KGs) that depend on the application context (115), we can define them as large, heterogeneous knowledgebases modeled through graphs and ontologies, which derive new knowledge from existing datasets (116). KGs are undergoing a renewed interest not only in academia but in the industry as well (117). In addition to storing structured, contextual data, the principal reasons are the capability of obtaining new conclusions from existing data through reasoning (118), and the possibility to enrich machine-learning models by providing context and produce extra information through derived measures or embedding strategies (119–124). Lastly, advances in machine learning create new opportunities for automating the construction and exploitation of biological KGs (125). We summarize several platforms that, due to their broad integrative scopes, can be seen as Biological Knowledge Graphs.

The Monarch Initiative (85) is an ambitious endeavor that uses an ontology-based strategy to deeply integrate genotype–phenotype data from many species and sources, enabling computational interrogation of disease models and revealing complex genotype–phenotype relationships. Monarch employs RDF to ingest a variety of external data sources, modeling several complex data types and connecting entities from different databases. SciGraph (https://github.com/SciGraph/SciGraph) is its central database engine, which provides means to represent ontologies and data described using ontologies as a Neo4j graph. The resulting combined corpus of graphs, from ontologies and ingested data, constitutes the Monarch Knowledge Graph. The platform provides several data access means for graph querying, application population and phenotype matching, as well as a web portal. The Monarch Web Portal (https://monarchinitiative.org/) exploits the graph to provide the users with several powerful features, in the likes of basic search, integrated information on entities of interest, search by phenotype profile, or text annotation.

Similarly, on a smaller scale, Pheno4J (75) provides a Java-based solution that loads annotated genetic variants and well-phenotyped patients into Neo4j. In order to build the database, Pheno4J requires user-generated files with the patient’s genetic variant and phenotype relations on the one hand, and both the Human Phenotype Ontology (HPO) (126) and a gene-to-HPO file on the other.

Focused on the analysis and discovery of comorbid diseases in humans, GenCoNet (127) proposes a semi-automatic pipeline that provides the import, fusion and analysis of stable disease, gene, variant and drug data in a Neo4j database, resulting in a KG for network analysis of gene–disease associations. The workflow consists of four concrete steps. The first step determines comorbidities of high interest and obtains Disease Ontologies terms associated with genes. Secondly, the workflow obtains genes associated with disease variants from HPO, MalaCards (128), DisGeNet (129) and OMIM (https://omim.org). The third step determines the gene controlled by eQTL and associated with the disease. Lastly, it finds the drugs, extracted from DrugBank (130), which target genes and treats or contraindicate the disease. GenCoNet showcases the KG by employing network analysis to detect drug-induced diseases or contraindications of drugs.

We can also find hybrid approaches that utilize different database implementations to build the KG (131). Canevet *et al*. build on the Ondex software platform (132) and employ both triple stores and the Neo4j, which supports gene-evidence graph patterns by making the KGs accessible via Cypher. The data integration is harmonized through the Bio-Knowledge Network Ontology (BioKNO), a lightweight and general ontology. Likewise, focused on bacterial whole-genome sequencing (WGS), Spfy (88) employs ontologies and different database paradigms to integrate disparate data sources and formats. Spfy primarily uses Blazegraph (https://blazegraph.com/) for storage along with MongoDB (https://www.mongodb.com/) to cache a hash table for duplicate checking, arguing a more efficient approach than would be possible through a search of the graph structure. The graph allows retrospective comparisons across stored results as more genomes are sequenced or populations change.

As mentioned before, ontological and semantic approaches have proved its utility in knowledge-intensive domains like the biomedical domain. Exploiting semantic and logic descriptions is natural for graph databases and triple stores and can be of great importance in KG implementations. In contrast to the rest of similar efforts, BioGrakn (133) builds upon Grakn (https://grakn.ai/) to deliver a KG with deductive reasoning capabilities. It employs almost the same data sources as BioGraphDB, but its model is designed through an ontology implemented in Graql, the Grankn’s declarative, knowledge-oriented graph query language. In the same vein as OWL and SWRL standards, Graql allows categorizing objects and relationships into distinct types, enabling inference and validation, used for searching genes linked to a particular Gene Ontology annotation, pathways linked to a particular gene, or finding all the upregulated differentially expressed (DE) miRNAs that also have validated mutations.

## Discussion

The literature body shows several advantages when biomedical systems and applications employ a graph model in the storage layer. The graph model is especially useful for representing and accessing biological data because path-based queries are intuitive in biological networks, closer to real-world conceptualizations. RDF schema or OWL Bio-ontologies easily translate into a graph because they are already based on triples, which can be further expanded by identifying implied relations between classes through logical reasoning (134). Also, exploiting graph theory algorithms and subgraph matching queries enables the inspection and discovering patterns of interest within the graph structure. GDBMSs schema-less/schema-optional grants a high degree of flexibility in research settings, allowing applications to adapt and evolve quickly and introduce abstraction and specialization of entities and relations among them more easily. This adaptability eases data integration tasks, as we have seen in many of the integrative platforms.

Specialized, industry-ready GDBMSs are relatively new and well-established biological systems build upon conventional databases, typically RDBMSs. Relevant examples are the protein databases (135), which have to deal with millions of protein/complex interactions, as is PPI databases’ case (136, 137). As described in the technical background, the underlying design of relational systems can lead to a trade-off between data integrity and performance. BioGRID (138), for instance, approaches this problem by utilizing a suite of tables specifically engineered to optimize query time while maintaining a structured normalized form that does not compromise fundamental design principles. Other relevant databases like DIP (139), IntAct (140) and STRING (141) maintain their relational model to fulfill the storage needs without further considerations concerning performance.

As seen in section 2 (43), Have and Jensen employed STRING as the use case to evaluate GDBMSs in biomedical settings and confront against RDBMSs, generally finding better performance of the former in usual tasks in the context of PPI networks. In section 3, we also see that many applications integrate PPI databases by explicitly transforming protein entries as nodes and intermediate relationship tables directly as edges, reporting performance improvements with GDBMSs over RDBMSs in some of the reviewed works (45, 87). Still, it is important to remark that redesigning the data storage/access layer usually involves a notable development effort, which may discourage research teams (usually short in human and economic resources). Since most of the protein databases are freely available, it would be relevant to compare their current implementation and a GDBMS implementation through formal benchmarks in that specific scenario, justifying or not engaging such development.

There exist limitations and potential issues of which developers need to be aware. While ontologies avoid designing specific problem-oriented data models and minimize reliability issues, these may increment the model’s complexity, jeopardizing the performance and integration time. If more relaxed schema approaches are adopted, the main trade-offs are deciding when certain data items become nodes or attributes and restraining both model complexity and integrity. Regarding performance, comparative benchmarks and more *adhoc* studies are quite heterogeneous and show disparate findings in some cases, making it challenging to identify a performance baseline to favor a concrete technology. Those focused on specific problems, like biological questions, report better GDBMS performances and qualitative features for managing networks (42, 43, 45, 47, 52, 55, 56). More formal benchmarks (50) and (52) report superior RDBMS results in several categories, especially for grouping, sorting, aggregating and setting operations. However, in graph analytics workloads that mainly consist of multi-table joining, pattern matching or path identification, GDBMSs still perform better. The gap widens as the size of the dataset increases. Yet, some benchmarks report problems when the graph is large. In the case of Neo4j, the number of edges to evaluate and subgraph pattern matching size may be a performance pit. This situation requires GDBMSs to provide proper mechanisms, like node replication or partitioning, or forego features like schema-less as TigerGraph does. All in all, GDBMSs are not necessarily superior in all graph queries, and, like any development, the aims and operational context should dictate the technological choices.

From a development point of view, big projects naturally tend to adopt traditional relational databases because they require industry-level tools and libraries that ensure code quality and architectural features such as scalability, integration and standard design patterns. Both industry and communities back RDBMS implementations with reliable frameworks that ease its adoption with, for instance, database to object abstraction layers. However, at this point, many current GDBMS implementations also offer proper frameworks, programming interfaces and Object-Graph Mapping that fulfill such needs.

Another important consideration is the current lack of standardization of query languages and data access methods across GDBMS implementations at both syntactic and theoretical levels (142). Apache Tinkerpop (https://tinkerpop.apache.org/) provides a high-level framework and the functional graph traversal language Gremlin, but not all GDSMS integrate it and this approach implies more coupling with the application code. Neo4j’s Cypher is a declarative language with similarities to common query languages and provides a clear graph path description syntax with full Create, Read, Update, Delete capabilities, making it one of the best solutions for graph querying. Cypher is the root of openCypher, a fully specified and open query language for property graph databases with >10 implementations across GDBMS solutions, even non-native ones like RedisGraph. TigerGraph follows a different approach with GSQL (https://docs.tigergraph.com/dev/gsql-ref), another powerful graph query language. It maintains backward compatibility with SQL, imposing a strict schema declaration in the query definition, and the queries behave as stored procedures, consisting of multiple SELECT clauses and imperative instructions such as branches and loops. This design targets enterprise applications, where the number and heterogeneity of external sources are not a concern, but instead, the size and performance, by optimizing storage format and query execution strategy, obtaining exciting results, as seen in Rusu and Huang (51). Fortunately, at the time of writing, the international committees that develop the SQL standard have voted to initiate *Graph Query Language* (GQL) (https://www.gqlstandards.org/) and intend to develop a declarative graph query language that builds on the foundations of SQL and integrates proven ideas from the existing openCypher, Oracle’s PGQL, GSQL and G-CORE (143) languages, a move that ensures the future of GDBMSs.

We have seen different technologies come and go, and deciding a GDBMS that satisfy the necessities may also become a time-consuming task that can be seen as five steps or stages: problem analysis, requirements analysis, GDBMS analysis, benchmarking and GDBMS selection (144). Sites like https://db-engines.com provide useful ranks, comparative tables and insights that help in the selection process. From what we have seen in the literature, Neo4j outstands in its adoption, not only in the biomedical domain, mainly due to the powerful Cypher query language, decent performance and ease of implementation. We foresee that this situation will be less evident in the near future, given the number of competitive developments in the field.

## Conclusion

In this work, we have followed the evolution and current landscape of GDBMSs, reviewed the bibliography looking for methods to evaluate their performance in different contexts and explored their applications in the biomedical domain. While RDBMSs and other NoSQL engines still provide better scalability options, more standardized query languages and more efficiency on typical data aggregation operations, most of the comparative analyses note that their performance suffers in densely connected datasets that imply a majority of many-to-many relations. Scenarios with a significant volume of complex relationships may benefit from GDBMSs for the following reasons: (i) graphs provide more natural modeling of many-to-many relationships; (ii) graph-oriented query languages provide more intuitive means for writing complex network traversal and graph algorithm queries than table-oriented ones like SQL, which require to join tables explicitly and reference columns; (iii) the schema-less/optional grants flexibility and (iv) in most situations, GDBMSs present higher performance for relationship-centric searches, like path traversals. These features yield several advantages for the biomedical domain, like easing the communication between domain experts, providing tools for discovering entities/clusters/patterns within the graph structure and facilitating data integration tasks, all of them very common when the investigation involves multiple sub-domains.

GDBMS technology is rapidly evolving to tackle scalability and similar operational weaknesses, offering a wide range of reliable choices to support the storage layer for either small prototypes or large, production-ready projects. The collection of described use cases and author experiences provides evidence that GDBMSs are very fit for biomedical data, as an individual storage system or as part of a hybrid, partitioned architecture. Moreover, by providing direct access to a graph model, late GDBMSs enable the use of graph algorithms and analytics in a very transparent way, improving hypothesis generation and testing.
